# Optiknot 3D—Free-Formed Frameworks out of Wood with Mass Customized Knots Produced by FFF Additive Manufactured Polymers: Experimental Investigations, Design Approach and Construction of a Prototype

**DOI:** 10.3390/polym12040965

**Published:** 2020-04-21

**Authors:** Benjamin Kromoser, Thomas Pachner

**Affiliations:** 1 Institute of Structural Engineering, University of Natural Resources and Life Sciences, Peter-Jordan-Straße 82, 1190 Vienna, Austria; 2 Pachner GmbH, 4710 Grieskirchen, Austria

**Keywords:** sustainable engineering, resource-efficient building construction, parametric design, additive manufacturing, 3D printing, mass customization, Fused Filament Fabrication (FFF), freeform design, wood construction, wooden shells

## Abstract

Free-formed frameworks are architecturally appealing constructions. They allow for maximum creative freedom as well as for a structural optimization of the support structure. The design and construction of these kind of structures is complex however, and therefore challenging, with each frame member having an individual length, each cladding plate an individual dimension and especially each knot having an individual geometry. The result is that geometry optimization and production technology become the most important processes when striving for an economic and ecological construction. The goals of the authors are the automation of the design process by applying a parametric model and the collection of the complete complexity in the knots as well as the production of these knots without material wastage by additive manufacturing. The development process was split into three different phases: (1) Preliminary experiments determining the tension, compression and bending load-bearing behavior of the knots produced by additive manufacturing, using different polymer-based materials: ABS, ASA, PA-CF, PA6CT, PCX, PETG and a mixture of PLA and ABS. (2) Development of an automated digital workflow for the design and production of these structures by the use of a parametric approach. (3) Design, production and assembly of a full-scale prototype in the form of a free-formed shell structure spanning an area of 20 m^2^. The prototype was made from fumed oak wood members in combination with white stained plywood panels connected by knots produced by Fused Filament Fabrication (FFF) additive manufacturing, using polymer-based materials and screws. At the end of the contribution, a summary and an outlook on further research is given.

## 1. Introduction

**Figure 1:**
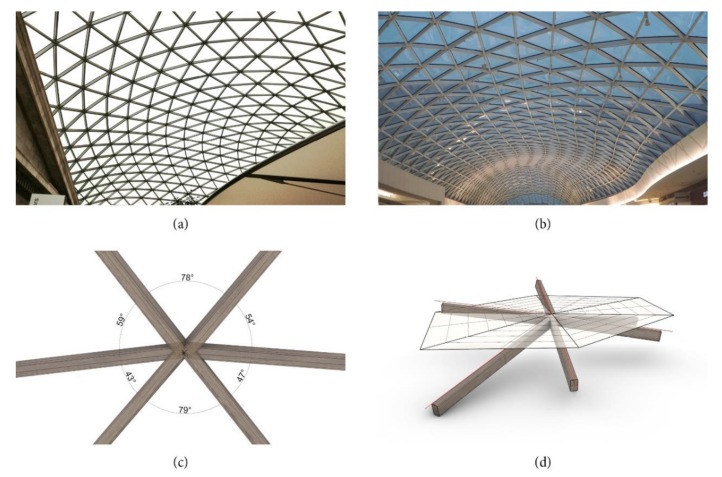
(**a**) British Museum Great Courtyard roof in London; (**b**) Eurovea Shopping Centre in Bratislava; (**c**,**d**) varying angles in space between the individual frame members that have to be considered for the design and production of the knot.

Free-formed building structures like the British Museum Great Court roof in London [1] (see Figure 1a) or the Eurovea Shopping Centre in Bratislava (see Figure 1b) constitute architectonically appealing building structures and have become an important part of modern architecture. The constructions are challenging from a geometric and technical point of view. Particularly demanding is the fact that the individual frame members all meet each other at different angles in space, as shown in Figure 1c,d. The consequence that arises from this is an individual design of each knot of the structure leading to a high complexity within the design and production. Available design and manufacturing possibilities are characterized by specific optical and technical properties, which limit the creative and technical potential. A general overview of the different knot designs/solutions is given in [2]. The solution described in [3], for example, represents a star-shaped knot that is visible from below. Within the production of this specific solution, all frame members have to be processed with a numerically controlled tube laser cutting machine. The frame members are afterwards welded to the star-shaped knot. In [4], on the other hand, a solution that allows one to connect tubular frame members with a knot consisting of multiple pieces is described. A special connection piece is mounted into the tubular frame member and is connected to a special spherical connection knot. The connection piece between the knot and the tube has to be slender to allow for adaptions to angles and, therefore, represents a potential weak point. In [5], a solution with an individualized topology optimized node for linking six cables in a strut, which was produced by additive manufacturing, is explained. The application to the addressed research is different as it represents a complex intersection between a strut and the tension members, but the basic approach is similar. Apart from that in [6], in which an approach to use individualized knots produced by additive manufacturing for facade construction is introduced. Again, the approach is to collect the complexity in the knot. In this case, knots for profiles meeting each other in different angles are introduced. The two last named solutions show the potential of mass customization in combination with additive manufacturing within the construction sector. One of the main mechanical requirements of a well thought out solution is an efficient load transfer which is characterized by a continuous load transfer (without redirecting the forces). This can be realized, for example, by continuous walls of the used profiles or by keeping the cross-section constant in the area of the knot. However, none of the available designs/solutions for the specific topic of connecting members for free-formed structures allow for an efficient load transfer in combination with a completely continuous appearance and an easy assembly.

Based on the current state-of-the-art, the aim of the presented research is the development of a digital process that enables an easy automated design and production of these kind of knots, using additive manufacturing. Substantial boundary conditions are, as stated above as currently non-available, an efficient load transfer, a waste-free production, a completely continuous design and an easy assembly.

The first research step, described in this paper, was to develop and test the full process, from design to production, of middle-sized wood frameworks with knots made from plastic created with Fused Filament Fabrication (FFF) additive manufacturing. The work, as presented subsequently, was split into three different phases: (1) Preliminary experiments of the load-bearing behavior of knots made from different kinds of polymer-based printing materials. (2) Development of an automated digital workflow by use of a parametric approach. (3) Design, production and assembly of a full-scale prototype in the form of a three-legged synclastic shell structure.

## 2. Basic Boundary Conditions and Development of the Connection between Node and Frame Member for an Efficient Load Transfer and an Easy Assembly

**Figure 2:**
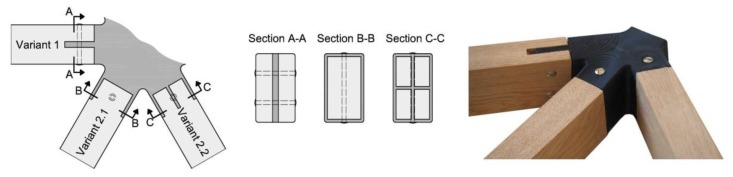
The three different variants for the connection between knot and frame member.

As defined above, the aim of the authors was to collect the entire complexity in the knot itself. Thus, the requirement was a simple as possible geometry of the frame members (constant cross-section, cut straight). Three different variants of the knot connections were designed and produced, as shown in Figure 2. The main feature of Variant 1 is the connection of the knot with the members using connecting tongues (mortise joints), ensuring simple assembly by the easy slide-in of the frame members from above or below, normal to the frame member axis. In addition to the improved construction, the dual screw connection ensures a high stiffness. A further advantage is that the screws can be tightened against wood, a ductile material, and not against the plastic of the knot. At this point, it has to be mentioned that the insert of the screw can become difficult if the angle between the frame members is small. The main feature of Variant 2.1 and 2.2 is a vertical and consequently easy screw connection. The assessment of the design showed that the frame members had to be inserted into the knot parallel to the axis of the wood member causing problems during assembly. Apart from that, both solutions demonstrated a comparatively low stiffness of the connection between knot and frame members. This was caused by a difficult to establish perfect fitting in practice between the knot and the wood member and a low lever arm between the center of the screw and the edge of the frame member. Due to the difficulty in establishing a perfect fitting and the fact that the screw can damage the plastic knot, we also decided to let the possibility of assembling the screws in Variant 2 in the same manner as in Variant 1 aside. After the first trail run, Variant 1 was selected for all further research and the experiments explained in Section 3.

## 3. Preliminary Experiments

### 3.1. Test Specimens and Configuration

Additive manufacturing technologies allow for an easy production of individual components with complex geometries. Beside the consideration of the raw production process, the possibility of lowering the environmental impact during the production of complex parts arises, as described in [7]. A recommendation for further research necessity regarding environmental implications like energy use, occupational health, waste, lifecycle impact, and crosscutting and policy issues can be found in [8]. Currently, elements produced by Fused Filament Fabrication (FFF) additive manufacturing are mainly used for applications with no or only few structural requirements in building construction (especially when thinking about constructive applications as part of the main supporting structure) and the testing procedures for the mechanical behavior focus on the testing of plastics [9,10,11,12,13]. The evaluation of the mechanical behavior (mainly just using small scale tensile tests) has been carried out in the course of several experimental investigations [14,15,16,17,18]. In [19], for example, especially the influence of different print layer orientations was investigated, resulting in a degradation in strength compared with bulk material properties (30–53%, depending on orientation) and as manufactured properties and as manufactured properties as reported by the FDM vendor (36–63%, depending on orientation). In [20], especially the balance between the bonding and deposition, taking especially the adhesive bond between the layers and the porosity as well as the interaction into consideration, were analyzed. Other research focuses on simulations and not actual experiments, as described in [21]. Studies show that the mechanical behavior depends on a large variety of parameters, for example, the chosen material, the printing parameters (temperature, extrusion rate, printing speed, layer thickness, nozzle diameter, etc.) [22], the layer orientation [23] or the infill type and grade. In addition to these parameters, it is also possible to improve the mechanical properties of a building component by intelligent design, as described in [24]. The work described in [25] focuses on the optimization of parts produced by additive manufacturing. In summary, it can be said that elements produced by (FFF) additive manufacturing have specific characteristics. These characteristics depend on a large variety of different parameters, which influence the load-bearing behavior, therefore making it difficult to predict when basing it on literature, especially when most of the described characterization are only based on tensile tests.

For these reasons, the authors decided to evaluate the load-bearing behavior of knots with a standard geometry printed from different plastic materials (ABS, ASA, PA-CF, PA6CT, PCX, PETG and a combination of PLA/ABS) in three types of full-scale tests: (1) tension tests by applying the force parallel to the frame member axis, (2) compression tests by also applying the force parallel to the frame member axis and (3) bending tests by applying the force normal to the strong axis of the frame members. The materials are commercially available and are partly modified from their raw starting material by the manufacturer. For example, PLA/ABS represents a mixture between PLA and ABS with the aim of improved properties. The high complexity of the structures for which the knots are designed leads to independent variations of load combinations (different load levels for each frame member an also for each knot) and a 1:1 reconstruction in experiments is therefore hardly feasible. The authors mainly expect compressive forces in a compression only shell in final construction state with different amount. The uniaxial loading under compression is assumed as a conservative assumption since a multiaxial compressive loading of the knot would lead to a multiaxial compression leading to a lower lateral stress and therefore a higher absorbable force. In addition, the bending and tension tests are performed to be able to evaluate a resistance in construction state. As proof of concept the chosen test setup gives a good overview how the interaction between manufacturing method (also with the chosen printer as well as the design) works in combination with the materials and which forces can be expected to be absorbed. The performance of standard tests would allow at this point of research no clear statement about the real behavior.

**Figure 3:**
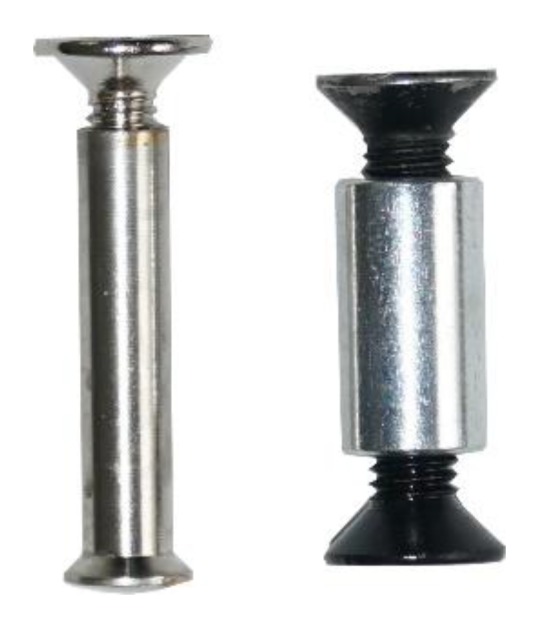
Used screws: (**left**) sleeve bolt (sb) and (**right**) a sleeve with screws from both sides (sl).

**Figure 4:**
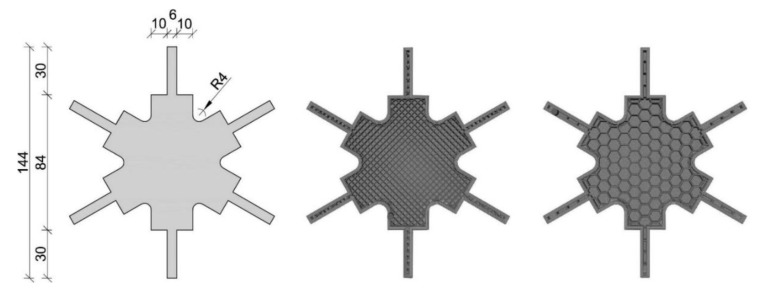
Top view of the design of the test knot (**left**), horizontal section of a specimen with 4 contour layers and 17% rectangular filling (**center**), horizontal section of a specimen with 4 contour layers and 17% honeycomb filling (**right**) [mm].

In total, 45 tests were performed (3 for PA6CT, 6 for ABS, ASA, PACF, PETG, PLA/ABS and 12 for PCX) in two test series. Variation parameters within the first series apart from the material such as (a) the number of bottom/top layers (3 or 4), (b) the number of contour layers (3 or 4) and (c) the infill grade (14% or 17%) were chosen. Within the second series, only the material PCX was tested while looking into the differences of infill type (rectangular and honeycomb), screw type (sleeve bolts and a sleeve with screws from both sides—see Figure 3) and the influence of using a nozzle with a higher diameter for extrusion in the 3D printer (diameter 0.8 mm) instead of a standard nozzle (diameter 0.4 mm). The authors focused on the use of standard infills with a view to an easy automation of the process. It would also be possible to adapt the infill according to the respective forces. The design of the test knot is shown in Figure 4 (left). In addition, a section of a specimen with four contour layers and 17% rectangular filling and a section of a specimen with four contour layers and 17% honeycomb filling are shown in Figure 4 (center and right). The holes for the screws were considered within the first print trials but it came up that this resulted in inaccuracies coming from the steadily varying geometry. Thus, it was decided not to print the holes and to drill them afterwards, in what led to a much better fitting. All configurations are listed in Table 1 for test series 1 and in Table 2 for test series 2. The used printing parameters are summarized in Table 3 and the material characteristics according to the manufacturers in Table 4. An industrial printer [26] with a heated chamber, two printing heads and material dryers were used for the production of all specimens (type: Xioneer Industrial). The material dryers are fix-mounted in the material holder of the printer and represent fans blowing hot air with a defined temperature at the printing material.

The manufacturing showed that the classic materials like ABS, ASA, PA6CT, PCX, PETG and the mixture of PLA/ABS are relatively good to process. In contrast, PACF is challenging to print since the raw material absorbs humidity (even under normal room temperature conditions). In addition, it is important to mention that within the production of the specimens (using all the material types), print jobs often failed midway, resulting in half-finished parts that had to be thrown away. The reason for these production fails can be traced back to software and hardware problems. The hardware problems were, for example, problems with the homogeneity of the material, feeding problems due to residues of the filament or a blocked nozzle. The software problems occurred, for example, due to problems within processing, which were due to the comparatively big dimensions (a finished knot weighing between 100 and 200 g) with long printing periods (often considerably exceeded 24 h) despite a high printing speed (up to 12,000 mm/min in *x*/*y* direction), leading to a high data volume, as well as problems due to an automated unsuitable printing path generation. The use of a bolt nozzle allowed for a reduction in the printing time of over 70% (for the test knots without support material).

### 3.2. Test Setups and Test Realization

**Figure 5:**
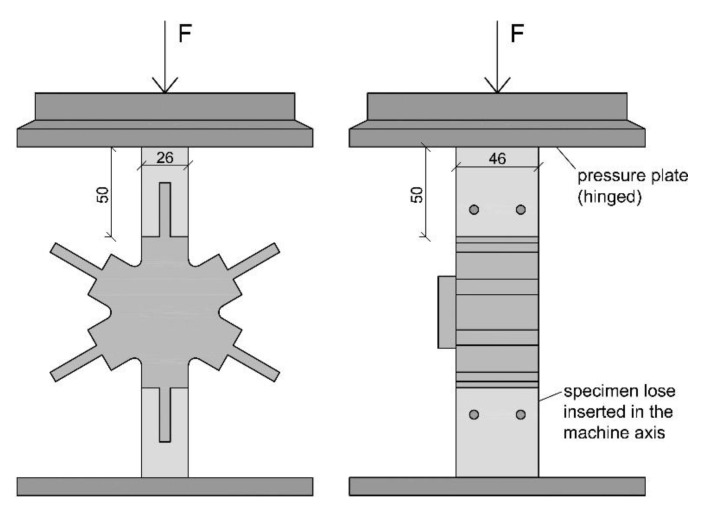
Test setup of the compression tests.

**Figure 6:**
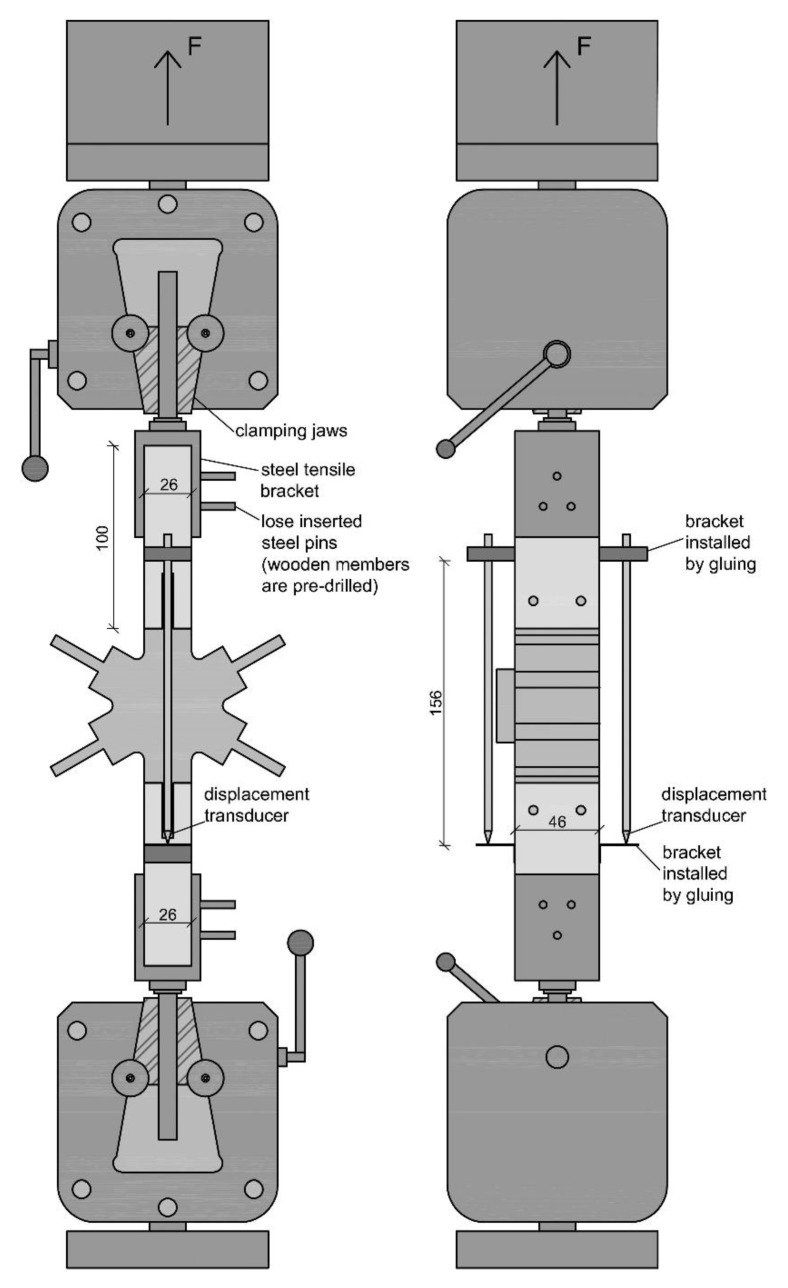
Test setup of the tension tests.

**Figure 7:**
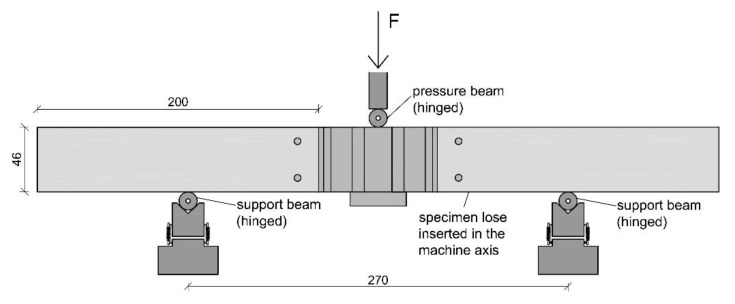
Test setup of the bending tests.

All tests were performed in a displacement-controlled manner. The feed rate was chosen with 1 mm/min for the compression tests and tension tests and 2 mm/min for the bending tests. As there is no standard regulating the feed rate, it was chosen according to experience from former tests and the expected duration of the tests. A servo-hydraulic testing machine with a nominal load of 63 kN (LFV 63-HH from Walter + BAI AG) was used to perform the experiments. For the compression and the bending tests, the forces were measured using an integrated load cell and the displacements using the machine integrated displacement transducer. During the tension tests, the displacements were determined by two additionally installed displacement transducers (same type) mounted by gluing the bracket for holding the device as well as a stop bracket to the test specimens (to the wooden frame members) with a measurement length of 156 mm. The displacement shown in the results represents the arithmetic mean of both. A special focus of the measuring concept was set on the interfaces between frame member and knot as well as the elongation of the knot itself. The reason for this is that the authors expect displacements between the wooden frame member and the knot. In addition, the stiffness of the knot is expected to be much lower than that of the wooden frame member. The test setup for the compression tests is shown in Figure 5, for the tensile tests in Figure 6 and for the bending tests in Figure 7.

### 3.3. Results of Test Series 1

#### 3.3.1. Results of the Compression Tests of Series 1

**Figure 8:**
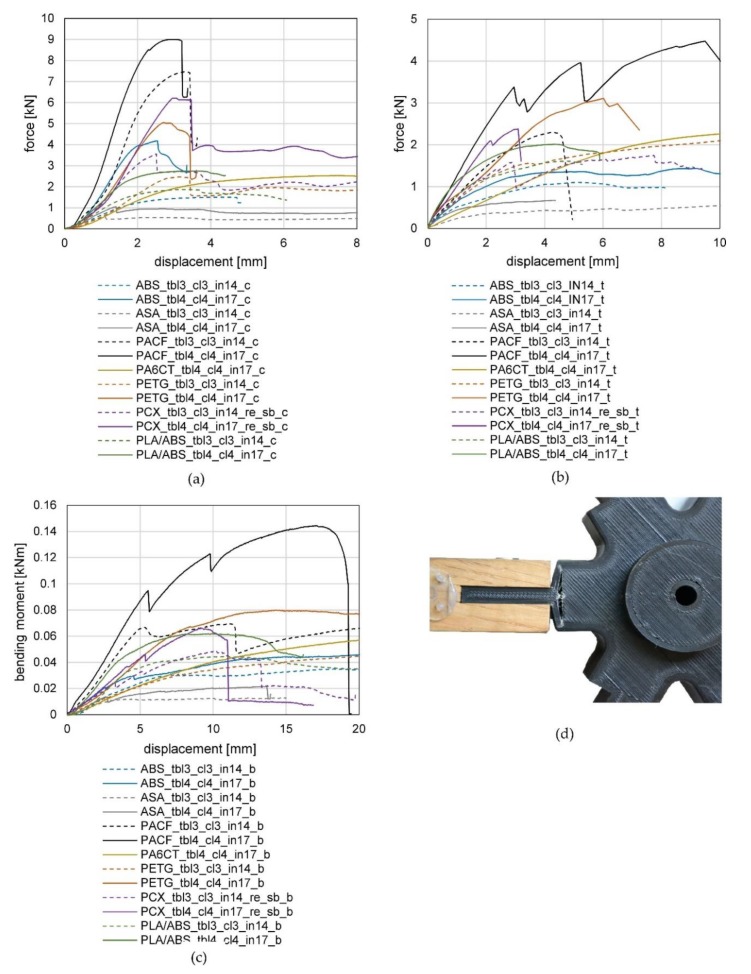
Results of the (**a**) compression tests; (**b**) tension tests; (**c**) bending tests, of series 1 and (**d**) tension failure caused by a loosening from the contour layers from the infill.

Firstly, we want to point out that the interpretation is based on one test per configuration due to the high number of variations. The test was repeated with another specimen if a discontinuity appeared in the results. The increasing stiffness at the beginning of the test can be explained by the fact that it takes time until the wooden frame members have full contact to the knot. The force–displacement curves of the compression tests of series 1 are shown in Figure 8a. An increase in the stiffness and maximum load-bearing capacity was observed during all tests for the specimens with four top/bottom/contour layers and the higher degree of filling (17%) in comparison with the specimen with three top/bottom/contour layers and 14% filling. The results show that PACF has, as expected, the best mechanical properties and a brittle failure behavior. The maximal forces for the two tested configurations were reached at 7.47 kN and 9.01 kN. A sudden spalling of the top and bottom layers of the knot (normal to the direction of loading) from the infill led to failure A similar failure behavior could be observed for PETG. The failure was not as brittle, but still a clear drop of the force could be observed during the spalling of the top and bottom layers. The maximal forces for the two tested configurations were reached at 2.47 kN and 5.06 kN. All other configurations showed a more ductile failure caused by a slow spalling of the top and bottom layer followed by a buckling of the detached layers and an accompanied compression of the infill. The maximum forces were reached for ABS at 1.51 kN and 4.19 kN, for ASA at 0.56 kN and 1.06 kN, for PCX at 3.56 kN and 6.22 kN, for PA6CT at 2.54 kN and for PLA/ABS at 1.87 kN and 2.75 kN.

#### 3.3.2. Results of the Tension Tests of Series 1

The force–displacement curves of the tension tests of series 1 are illustrated in Figure 8b. Similar to the results of the compression tests, an increase in the stiffness and maximum load-bearing capacity was observed for the specimens with four top/bottom/contour layers and a higher degree of filling (17%) in comparison to the specimen with three top/bottom/contour layers and 14% filling. As was the case during the compression tests, the PACF specimens showed the best results by reaching 2.30 kN and 4.47 kN maximum force. The drops in the curve of the specimen PACF_tbl4_cl4_in17_t result from the piecewise lifting-off of the contour layers from the infill (see Figure 8d). The fracture behavior for both PACF test specimens was brittle, with a tearing-off of the tongue leading to failure. A similar behavior could be observed for both specimens made from PETG, which also both failed by detachment of the tongue, with maximum loads of 2.53 kN and 3.11 kN. For all other materials, a slow loosening of the contour layers from the infill and a subsequent elongation of the interface between the knot and tongue led to failure. The maximum forces were 1.1 kN and 1.43 kN for ABS, 0.62 kN and 0.67 kN for ASA, 1.74 kN and 2.38 kN for PCX, 2.61 kN for PA6CT and 1.61 kN as well as 2.00 kN for PLA/ABS.

#### 3.3.3. Results of the Bending Tests of Series 1

The bending moment–displacement curves of the bending tests of series 1 are shown in Figure 8c. As observed during the tension and compression tests, the specimens with four top/bottom/contour layers and a higher degree of filling (17%) demonstrated an increased stiffness and maximum load-bearing capacity compared with the specimen with three top/bottom/contour layers and 14% filling. The fracture behavior for all tests was ductile. The PACF specimens achieved yet again the best results by reaching 0.070 kNm and 0.144 kNm maximum bending moment. The drops in the curve of the specimen PACF_tbl4_cl4_in17_b can be traced back to the piecewise detachment of the contour layers from the infill at the load averted side (similar to the tension tests). In comparison with the compression and tension tests, the knots printed from PLA/ABS showed a better performance by reaching 0.045 kNm and 0.063 kNm maximum bending moment. The maximum bending moments for ABS were 0.047 kNm and 0.036 kNm, for ASA 0.014 kNm and 0.022 kNm, for PCX 0.049 kNm and 0.067 kNm, for PA6CT 0.062 kNm and for PETG 0.049 kNm and 0.080 kNm. As failure modes, a combination of the failures observed within the compression and tension tests occurred at the compression side and tension side (in Figure 7 top edge and lower edge of the specimen). The stiff materials (PACF and PETG) failed brittle by tearing of the tongue initiated at the load averted side. All other materials failed ductile by either a shedding of the top layer at the upper surface (load facing side) with a subsequent buckling of the layer or by a loosening of the contour layers at the bottom of the knot (similar to the failure shown in Figure 10) followed by a separation of the tongue.

### 3.4. Results of Test Series 2

#### 3.4.1. Results of the Compression Tests of Series 2

**Figure 9:**
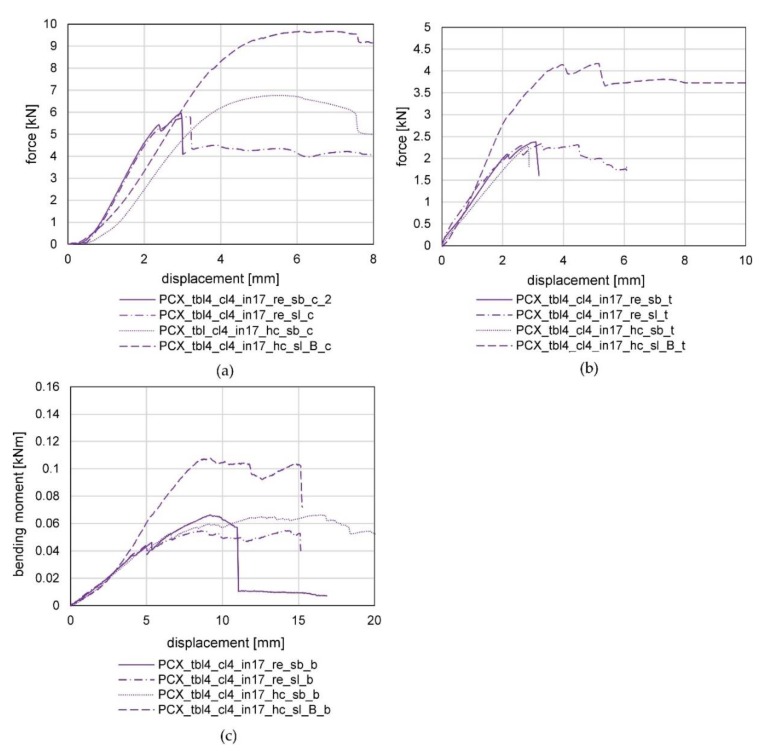
Results of the (**a**) compression tests; (**b**) tension tests; (**c**) bending tests, of series 2.

The test results of the compression tests of series 2 are depicted in Figure 9a. A comparison of the used screws (configuration PCX_tbl4_cl4_in17_re_sb_c and PCX_tbl4_cl4_in17_re_sl_c) confirms the assumption of their minor influence on the compression behavior (maximum forces 5.70 kN and 5.82 kN). This can be led back to the direct contact between the knot and the wood pieces. When looking at the infill type, the honeycomb showcased a lower stiffness but a higher maximum force (maximum force of PCX_tbl4_cl4_in17_hc_c 6.77 kN) than the rectangular infill. The lower stiffness can be explained by the irregular infill walls in comparison with the straight walls of the rectangular infill. A significant increase in the maximum load-bearing behavior could be achieved through the use of a 3D printer nozzle with a higher diameter (maximum force 9.68 kN). In this context, it must be mentioned that the increase in the nozzle diameter by keeping the number of top and bottom layers as well as the contour layers equal to the other specimens led to almost a doubling of the material usage.

#### 3.4.2. Results of the Tension Tests of Series 2

The test results of the tensile tests of series 2 are featured in Figure 9b. Contrary to the expectations, no influence of the screw type nor the type of infill on the load-bearing behavior of the specimens can be observed. The reason for the low influence of the screw type can be found in the sufficient strength of both types of screws, regarding their shear action and sufficient resistance against bearing stress of the wooden frame member as well as all the plastic knots. The standard configuration (PCX_tbl4_cl4_in17_re_sb_t) failed at a maximum load of 2.38 kN, the configuration with a sleeve and two screws (PCX_tbl_cl4_in17_re_sl_t) failed at 2.35 kN and the configuration with a honeycomb infill (PCX_tbl4_cl4_in17_hc_sb_t) failed at 2.28 kN. The use of a nozzle with increased diameter (diameter 0.8 mm) led to a significant increase in the maximal load-bearing capacity to 4.17 kN, resulting from the larger widths of the layers (see Table 3).

#### 3.4.3. Results of the Bending Tests of Series 2

The bending moment–displacement curves of the bending tests of series 2 are displayed in Figure 9c. Similar to the results of the tensile tests, only a minor influence of the screw type and infill type can be observed. The standard configuration (PCX_tbl4_cl4_in17_re_sb_b) reached a maximum bending moment of 0.067 kNm, the configuration with a sleeve and two screws (PCX_tbl4_cl4_in17_re_sl_b) 0.056 kNm and the configuration with a honeycomb filling (PCX_tbl4_cl4_in17_hc_sb_b) 0.068 kNm. As expected, the knot printed with the bolt nozzle reached a significantly higher maximum bending moment of 0.109 kNm.

### 3.5. Analysis of the Production of the Test Specimens and the Experiments

The tests showed that the knots can withstand a considerable static load under tensile, bending and especially under compressive loading within a short period of time. The static calculations within the design of the pavilion, which will be explained in more detail in Section 5, resulted in a maximum compression load of 3 kN per frame member, under the consideration of safety factors according to Eurocode. Thus, the basic functionality could be proven for the materials PACF and PCX for both tested configurations and for PETG as well as for ABS for the stronger design. The stronger knot construction out of PLA/ABS failed just below 3 kN, reaching a maximum load of 2.75 kN. PACF, being the authors first choice for indoor use (nylon is not resistant to ultraviolet (UV) rays), was very unstable during the printing process due to problems with high moisture levels and inhomogeneities in the material, and resulted in many misprints. If the process can be stabilized, PACF should be used for indoor applications for future projects. For outdoor applications, PCX is recommended due to its good mechanical properties, high resistance against UV-rays and other weather conditions, high chemical and heat resistance and foremost, its stable printing process. The experiments showed that not only the material type but also the number of top, bottom and contour layers as well as the infill grade, and consequently the amount of used material, have a big influence on the load-bearing capacity. Only a subordinate influence could be observed for the tested geometry within a variation of the screw and infill type. The authors initially expected problems due to high stresses at the hole face caused by the screws. Contrary to the expectations, no failures occurred for the chosen boundary conditions. During the compression tests, a lower stiffness and higher maximum load-bearing capacity could be observed for the infill type honeycomb. The use of a bolt nozzle with higher diameter (0.8 mm) instead of a standard nozzle (0.4 mm) showed great potential regarding the printing time, by reducing it by about 70% (for the standard knot without support material) without taking any noticeable toll on the surface quality.

## 4. Automated Design Approach for the Knots

**Figure 10:**
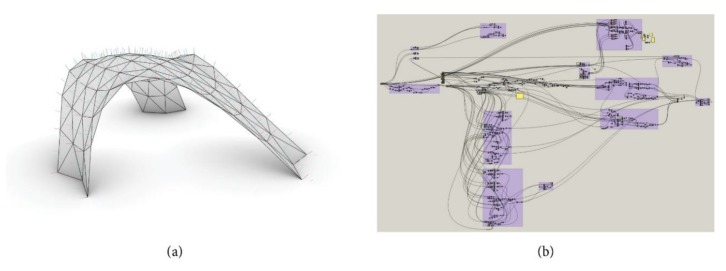
(**a**) Input geometry as basis for automated node design; (**b**) canvas of scripting interface.

The second phase of research contains the development of an automated digital workflow using a parametric approach. Each node geometry is generated automatically based on a predefined set of rules and varying input parameters. The basic geometry of the prototype is represented as a triangular mesh in combination with curves that determine the axis of knots and members (see Figure 10a). The shown structure is the result of the computational form optimization for the pavilion which will be described in Section 5 more detailed. This process represents the optimization of the global shape of the pavilion regarding an efficient load transfer. All information necessary to generate a full 3D model of the prototype was put into the parametric script, executed in Grasshopper [27] (Figure 10b, for an example of the script for the pavilion). Grasshopper is a visual programming language and environment that runs within the Rhinoceros 3D computer-aided design (CAD) application. Programs are created by dragging components onto a canvas. The outputs to these components are then connected to the inputs of subsequent components. Grasshopper is primarily used to build generative algorithms, such as for generative art. Many of Grasshopper’s components create 3D geometry. Programs may also contain other types of algorithms including numeric, textual, audio-visual and haptic applications [27].

Once the input geometry was loaded into the parametric interface, the final shape of the elements was generated step by step by transforming and obtaining information with the help of the script. As a result, all 82 knots of the prototype were constructed in full detail, each of them with a unique design. Consecutively, the authors will show the principle of the automated node-design based on the design of the knots for the prototype.

**Figure 11:**
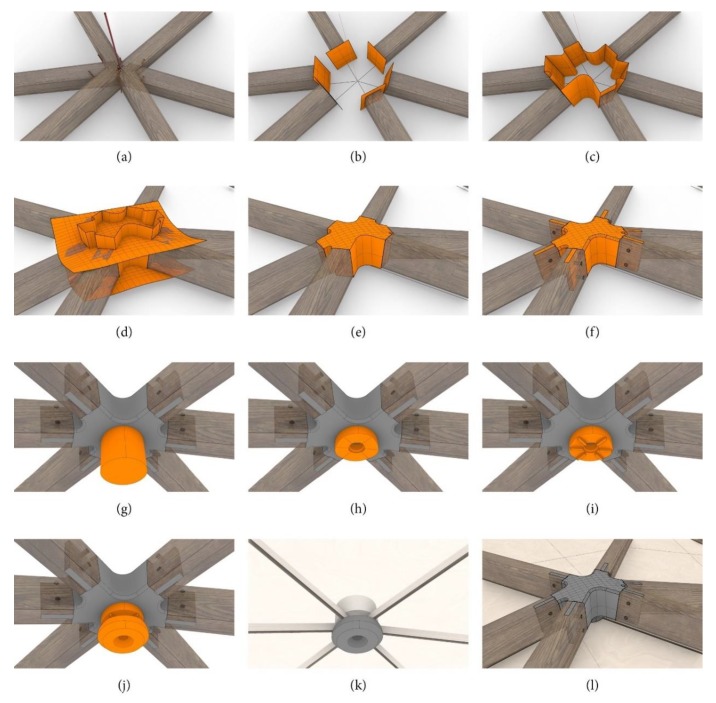
(**a**) Frame member intersection; (**b**) cutting planes; (**c**) lateral area of the knot; (**d**) patched surfaces; (**e**) basic knot geometry; (**f**) knot with connecting tongues (mortise joints) joining the frame members; (**g**) cylinder parallel to knot axis; (**h**) adapter on the inside of the knot 1st step; (**i**) finished adapter on the inside of the knot; (**j**) bracket for the fixation of the panels; (**k**) panel fixation detail; (**l**) final appearance from outside.

The first step of the design process is making the decision whether the frame structure should be on the inside or the outside of the mesh. In the case of the prototype, an outer positioning of the framework was chosen. The member cross-section is subsequently determined by the structural analysis of the respective construction. The distance from the lower bound of the member to the edges of the mesh has to be set to a fix value while simultaneously being influenced by the maximum kink angle of the two aligned faces of the mesh. After generating the position of each member in space, their intersections at the knots can be calculated (see Figure 11a). Each member has two neighbors and, therefore, two intersection curves. Since the authors decided to put all complexity in the knot and cut all members perpendicular to their axes, the cutting plane of each member depends on the neighboring intersection curves. Figure 11b shows these planes and the cut members for an exemplary knot of the prototype.

As it is the designs intent to generate a framework that fades from member to knot to member as continuously as possible, a filleting of the edges of the two aligned lateral faces is aimed for. The filleting radius is chosen automatically based on the angle between the members. A smaller angle requires a smaller radius. In the next step, the fillets and the cutting planes are connected, inserting plane surfaces, and thus closing the lateral area of the knot (Figure 11c). Polysurfaces are extended over the height of the members. The axes of members have varying angles to the axis of the knot, forcing the top and bottom surfaces of the knot to be free-formed in order to generate a flush connection to each member and a smooth appearance. This is achieved by using two patch surfaces with predefined spans and flexibility (Figure 11d). The Boolean intersection of the lateral area and the patch surfaces for the top and bottom surfaces connect the structure to a closed polysurface acting as the base geometry of the knot (Figure 11e). The edges of the knots are then filleted with a defined radius adjusted to the radius of the frame members. The addition of the connecting tongue (mortise joint) between the frame member and knot is part of the last design step, as shown in Figure 11f.

As it is the goal to put all the complexity in the knot, the panel fixation adapter is also attached to the knot. A cylinder with a fixed diameter is generated parallel to the knot axis (Figure 11g). The knot axis was defined as the dot product of the surface normal vectors of the adjacent areas. It is cut with planes, coplanar to the panels resting on the adapter (see Figure 11h). The panels are fixed to the framework with a constant parallel gap of 10 mm predefined by the spacer placed directly on the adapter. The gap was defined by the minimum distance for avoiding an intersection of the edges of the wooden frame member with the panels. A hole for the subsequent attachment of a metal bush for the fixation of the panels is included (see Figure 11i).

It is very important that the panels are clamped to the framework in a tight and durable manner. To ensure this, the brackets, which are a counterpart to the adapter, are also custom made (Figure 11j). With the attachment system complete, the panels can finally be fixed to the framework; only a single adequately dimensioned screw is needed (see Figure 11k,l). This allows for a simple and fast assembly and disassembly. As stated above, the panels are also individualized, with each having its own unique triangular shape. Despite the differences in size, the authors achieved a uniform appearance of the panels, from both the inside and outside, with a constant gap space and parallel edges. In order to ensure the perfect interaction between the frame members, the knots and the panels, the generation of the frame member length and the panels was also implemented in the script. Each panel is a polysurface, generated from various intersecting planes. To obtain the lateral surfaces of the panel, the plane that determines the orientation of the members is offset by the half of the desired gap between two neighboring panels. The top and bottom surface of the panels are created with the plane perpendicular to each face normal to the base mesh and a constant offset that determines the panel thickness. In order to avoid intersections with the bolt that fixes the panels to the knots, a Boolean difference is executed at each corner of the panels.

## 5. Design and Construction of the Full-Scale Prototype

### 5.1. Design

The main objective for the construction of the subsequently presented pavilion was to prove the functionality of the entire process, from the design to manufacturing. To the authors’ experience, this step is very important for the evaluation of the research since many challenges appear within the realization of a first prototype. The design was based on the idea of building a highly efficient structural system regarding material use in form of a free-formed compression-only shell. The authors used a computational form-finding method, already successfully applied for designing concrete shells [28], to determine the shape for the pavilion. Within the environment of a dynamic particle–spring system, a base mesh [29], resembling the footprint of the final shell, is constructed. Each nodal point of the mesh represents a particle. One or more forces transform the geometry of the elastic mesh into its final form. For the present optimization, the self-weight of the structure was applied. The deformed mesh geometry is a result of the equilibrium state of the particles due to the acting forces. This state is calculated using a physics-based geometric modeling approach realized with Kangaroo [30], the CAD environment Rhinoceros [31] and its plug-in, Grasshopper [27]. The pre-dimensioning for the pavilion was done by use of Karamba3d [32], a plug-in for Rhinoceros, and the final calculations were performed by implementation of a frame analysis resulting in normal forces of the 213 frame members by use of a commercial finite element software (Rstab from Dlubal). As the only load case, self-weight was considered as the structure was designed for indoor use. The self-weight was considered by the program itself for the frame members and the load of the panels was considered as line load at the frame members. Within the calculation all of the safety factors, according to Eurocode 0 [33] on the load side and Eurocode 5 [34] on the resistance side, showed that the computationally found design allowed for a maximum utilization of the wooden frame members of 61%. The maximum normal force was determined with 1.08 kN. The authors tripled this value to 3kN as requirement for the knots due to the unknown continuous load bearing behaviour of the knots and missing knowledge and partially safety factors for plastics in structural engineering.

**Figure 12:**
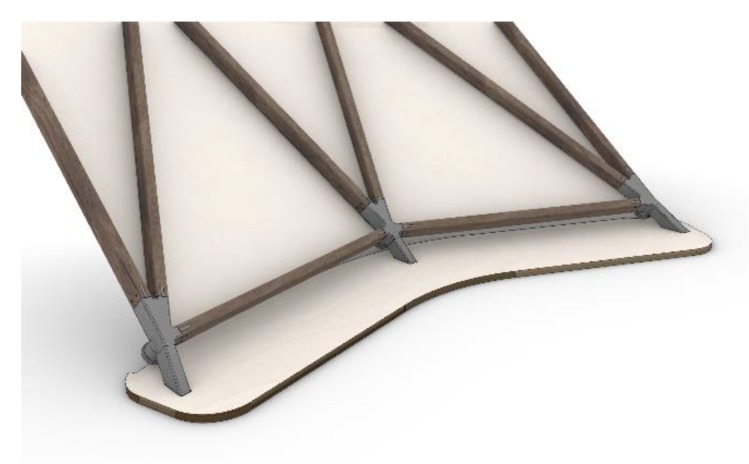
Fixation at the plywood plate which are fixed at the bottom.

The 213 frame members (cross-section: 26 × 46 mm), which formed the external arranged supporting structure, were made of smoked oak. Regarding the connection between the knot and wooden frame members, variant 1 was chosen with screws of the type (sl). The 144 panels, each with an individual geometry and made from 8 mm stained birch plywood, were mounted to the inside of the structure as cladding. They were formatted by a 5-axis computer numerical controlled (CNC) mill (Homag centateq P-300, CAM software woodWOP) allowing for parallel edges, based on the defined script described in Section 4. The 5-axis mill was required because of the edge angles, which were individual for each edge of each panel (required for a continuous parallel joint). As previously mentioned, the fixing apparatus was constructed as part of the knot, taking the individual geometry of the panels with integrated spacers into consideration. A steel threaded socket was screwed into the knot and allowed for an easy fixation with one 8 mm diameter countersunk head screw and a special adapter, which was the exact counterpart to each knot and had also been produced by additive manufacturing. In total, 82 individual knots were integrated in the pavilion. Nine of them were used as connection pieces to the plywood plates used for the fixation of the structure to the ground (see Figure 12). Initially, PACF was chosen as the production material for the knots. Due to problems within the printing process, ABS and PLA/ABS with higher top and bottom layers (five) and a higher infill (20%) were looked into as the material of choice. In the end, the final knots were made of PCX in order to test this material for further applications. The total weight of the pavilion amounted to 280 kg.

### 5.2. Assembly

**Figure 13:**
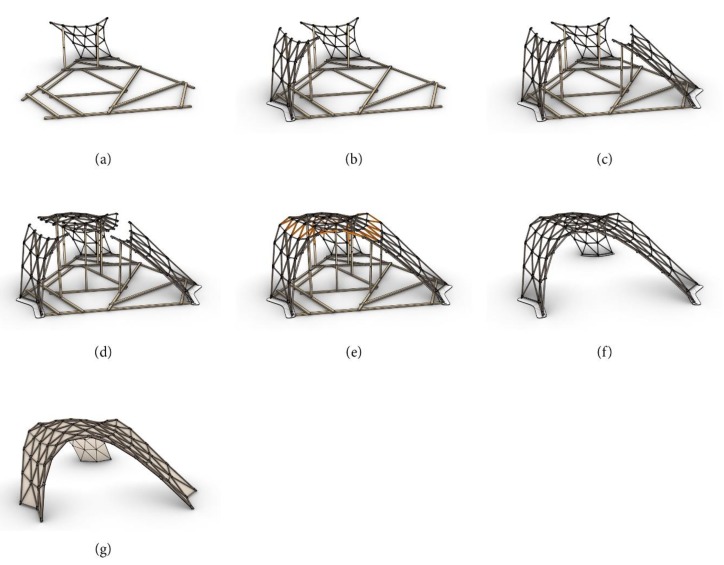
Assembly of the pavilion (**a**) ready assembled falsework and assembly of supersize element 1; (**b**) assembly of supersize element 2; (**c**) assembly of supersize element 3; (**d**) assembly of supersize element 4; (**e**) assembly of the loose members (orange); (**f**) removal of the falsework; (**g**) mounting of the panels.

**Figure 14:**
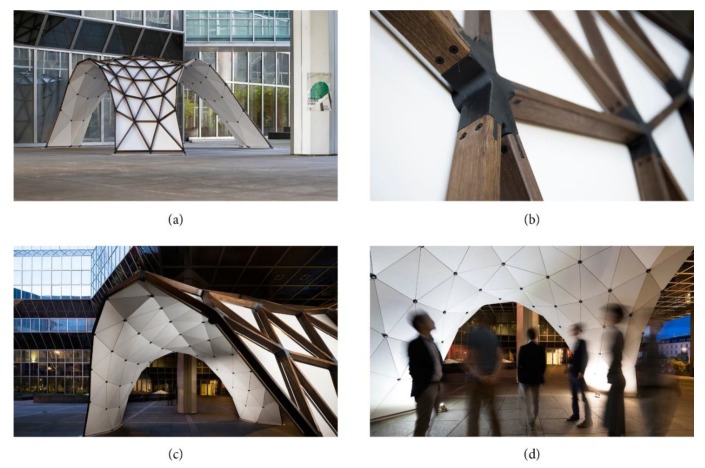
Prototype Optiknot 3D © Christoph Panzer: (**a**) View from outside; (**b**) knot detail; (**c**) evening view; (**d**) view from inside.

The pavilion was prefabricated in four supersize elements, as shown in Figure 13. A special falsework, made of spruce square timber with the dimensions of 50 × 80 mm and total weight of 200 kg, was used to support the individual elements during the final connection with the three rows of loose members (see Figure 13e in orange). A ball joint was used for the support points. The focus of the authors was set on developing a lightweight supporting system that could easily be assembled and reused, since usually falsework required for the construction of shells has a very high material demand. Once the grid shell was completed, the falsework was removed and the cladding panels, beginning from the bottom part of the structure and ending at the vertex, were mounted. A visualization of the finished structure is shown in Figure 13g and photographs of the actual built structure are shown in Figure 14a–d.

## 6. Summary

The design and construction of free-formed frameworks is challenging and complex. The reason for this is that these kinds of structures are characterized by an individual geometry of each frame member, cladding plate and particularly of each knot. The objectives of the authors were the automation of the design and production process by use of a parametric model, the collection of the complete complexity of the structure in the knots and a waste-free production of these by additive manufacturing. The development process was split in three different phases.

First, preliminary experiments concerning the tension, compression and bending load-bearing behavior of the knots, printed in different configurations from the materials ABS, ASA, PACF, PA6CT, PCX, PETG and PLA/ABS, were performed. The study showed that carbon fiber reinforced nylon (PACF) has the best mechanical properties but unfortunately provides a low resistance against UV-rays and causes difficulties within the printing process. The experiments also showed that ABS and PETG are suitable materials regarding the mechanical behavior. ASA, which would have a high resistance against UV-rays and other climatic influences, showed the worst mechanical behavior and is not suitable for constructive applications. Beside PACF, the material PCX showed the best mechanical properties while simultaneously being resistant to UV-rays and other climatic influences. Looking at all of the results, the authors recommend the use of PACF for indoor applications, once the printing process is stabilized, and PCX for outdoor applications. The experiments also showed that the tested screw types as well as the tested infill types (rectangular and honeycomb) only have a minor influence on the load-bearing behavior and can, therefore, be neglected. However, it would be alternatively possible to adapt the infill individually according to the occurring forces. The potential would have to be tested in further research work. Great capability was seen when using a bolt nozzle with a higher diameter as the printing time was reduced by about 70% and a significant increase of the load-bearing capacity, ascribed to the elevated material usage, was noted. A clear correlation between the amount of used material and the load-bearing capacity could be observed, with a high number of top/bottom/contour layers as well as a higher infill leading to a direct increase.

The second phase consisted of the automation of the digital workflow by the use of a parametric approach. It was found that the optimization underlies several boundary conditions, such as the very strong distortions and varying angles between the frame members. Nonetheless, the process was developed and stabilized, and the functionality was proven for a first prototypical application designing a free-formed wooden framework with plastic knots printed by FFF additive manufacturing.

In the third step, a prototype pavilion was successfully designed (by use of computational form finding) and was assembled successfully. The manufacturing of the pavilion showed that the very lightweight structure had a comparatively low stiffness. A possible improvement would be an increase in the counter-curvature at the legs of the structure and an increase in the counter-curvature of the rim. Up to the current knowledge, an upscaling with the same detailing is possible but limited due to the mechanical properties of the good printable polymers. Especially, the behavior under cyclic loading (for example, wind) and the creep behavior are issues which have to be investigated in more detail. However, the authors strive to consider an upscaling to a factor of two of the presented pavilion with the presented approach and will focus at using metal parts (with 3D metal printing as the technology) for structures with bigger dimensions than that.

Further research work of the authors on the structural aspects will focus on the construction of sealed weather resistant structures made out of wood with plastic knots. Apart from that, the evaluation of further layer numbers and infill grades for the material PCX as well as the long-term behavior of the various plastic materials will be addressed. The long-term vision is to further the development of the process for the application of steel–glass constructions.

## Figures and Tables

**Table 1:** Variation parameters test series 1 (infill rectangular).

Specimen Designation	Material	Top/Bottom Layers	Contour Layers	Infill Percentage
ABS_tbl3_cl3_in14	ABS	3	3	14
ABS_tbl4_cl4_in17	ABS	4	4	17
ASA_tbl3_cl3_in14	ASA	3	3	14
ASA_tbl4_cl4_in17	ASA	4	4	17
PACF_tbl3_cl3_in14	PACF	3	3	14
PACF_tbl4_cl4_in17	PACF	4	4	17
PA6CT_tbl4_cl4_in17	PA6CT	4	4	17
PETG_tbl3_cl3_in14	PETG	3	3	14
PETG_tbl4_cl4_in17	PETG	4	4	17
PCX_tbl3_cl3_in14	PCX	3	3	14
PCX_tbl4_cl4_in17	PCX	4	4	17
PLA/ABS_tbl3_cl3_in14	PLA/ABS	3	3	14
PLA/ABS_tbl4_cl4_in17	PLA/ABS	4	4	17

**Table 2:** Variation parameters test series 2.

Specimen Designation	Material	Top/Bottom Layers	Contour Layers	Infill [%]	Infill Type	Screw Type
PCX_tbl4_cl4_in17_re_sb	PCX	4	4	17	rectangular	sleeve bolts
PCX_tbl4_cl4_in17_re_sl	PCX	4	4	17	rectangular	sleeve w. screws
PCX_tbl4_cl4_in17_hc_sb	PCX	4	4	17	honeycomb	sleeve bolts
PCX_tbl4_cl4_in17_hc_sl_B	PCX	4	4	17	honeycomb	sleeve w. screws
PCX_tbl4_cl4_in17_re_sb	PCX	4	4	17	rectangular	sleeve bolts
PCX_tbl4_cl4_in17_re_sl	PCX	4	4	17	rectangular	sleeve w. screws
PCX_tbl4_cl4_in17_hc_sb	PCX	4	4	17	honeycomb	sleeve bolts
PCX_tbl4_cl4_in17_hc_sl_B	PCX	4	4	17	honeycomb	sleeve w. screws

**Table 3:** Printing properties (s: standard nozzle, b: bolt nozzle).

Material	Layer Height [mm]	Layer Widths [mm]	Nozzle Diameter [mm]	Chamber Temperature [°C]	Extrusion Temperature [°C]	Bed Temperature [°C]	Standard Printing Speed [mm/min]
ABS_s	0.2	0.504	0.4	60	245	90	3000
ASA_s	0.2	0.528	0.4	60	265	90	3300
PACF_s	0.2	0.792	0.6	70	270	100	2400
PA6CT_s	0.2	0.528	0.4	50	260	60	3000
PETG_s	0.2	0.552	0.4	40	255	60	3600
PCX_s	0.2	0.504	0.4	70	260	105	3600
PCX_b	0.5	1.056	0.8	70	260	105	2100

**Table 4:** Material characteristics (manufacturer specifications).

Material	UV Resist.	Water Resist.	Chemical Resist.	Tensile Strength at Yield (ISO 527) [MPa]	Tensile Modulus (ISO 527) (MPa)	Density (ISO 1183) [g/cm^3^]	Strain at Break [%]	Heat Deflection Temp. [°C]
ABS	no	-	no	35.0	2030	1.03	8	103
ASA	yes	yes	-	47.5	2020	1.11	15	98
PACF	-	-	-	107.0	-	1.40	2	120
PA6CT	-	-	-	66.2	2223	1.12	10	67
PETG	-	-	-	50.0	2020	1.27	23	70
PCX	yes	-	-	59.7	2048	1.19	12.2	113
PLA/ABS	no	-	no	40.0	4000	1.27	47	95

## Abbreviations

Tbl	top and bottom layer
cl	contour layer
in	infill
c	compression
t	tension
b	bending
ABS	Acrylonitrile butadiene styrene-based material
ASA	Acrylic styrene acrylonitrile-based material
PACF	Advanced carbon fiber infused polyamide-based material
PA6CT	Polyamide-6/6,6-copolymer-based material
PCX	Polycarbonate-based material
PETG	Polyethylene terephthalate (PET) modified with glycol
PLA	Polylactide
